# Crimean-Congo hemorrhagic fever virus Africa 1 lineage in *Hyalomma* species ticks, Tunisia, 2024

**DOI:** 10.1128/jvi.01401-25

**Published:** 2025-09-23

**Authors:** Silvia Fabi, Giuseppe Di Pietra, Elisabetta Valente, Massimo Bellato, Chiara Agostini, Michele Paccagnella, Elisa Franchin, Valentina Chisu, Cipriano Foxi, Claudia Del Vecchio, Ignazio Castagliuolo, Giovanna Masala, Cristiano Salata

**Affiliations:** 1 Department of Molecular Medicine, University of Padova 208970, Padua, Italy; 2 Istituto Universitario di Studi Superiori, Pavia, Italy; 3 UOC Medicina di Laboratorio, Ospedale San Bassano, Bassano, Italy; 4 Microbiology and Virology Diagnostic Unit, Padua University Hospital 18624, Padua, Italy; 5 Department of Animal Health, Istituto Zooprofilattico Sperimentale della Sardegna, Sassari, Italy; St Jude Children's Research Hospital, Memphis, Tennessee, USA

**Keywords:** *Hyalomma*, CCHFV, *Nairovirus*, Tunisia

## LETTER

Crimean-Congo hemorrhagic fever virus (CCHFV) represents the most widespread tick-borne high-consequence pathogen globally, having been detected across Africa, Eastern Europe, the Middle East, and Asia. Recently, CCHFV has gradually expanded its geographical distribution into southwestern Europe, including Spain, Portugal, and France (1–4). Phylogenetic analysis suggests multiple virus introductions, likely via migratory birds carrying infected ticks from Africa (1, 3, 4).

In this study, we investigated the presence of CCHFV in *Hyalomma* ticks collected in Tunisia, a country where CCHFV circulation has been documented in animals (5). Tunisia serves as a stopover site for migratory birds before they cross the Mediterranean Sea, representing a potential hotspot for the acquisition of infected ticks that may subsequently be released in succeeding stopover sites.

**Fig 1:**
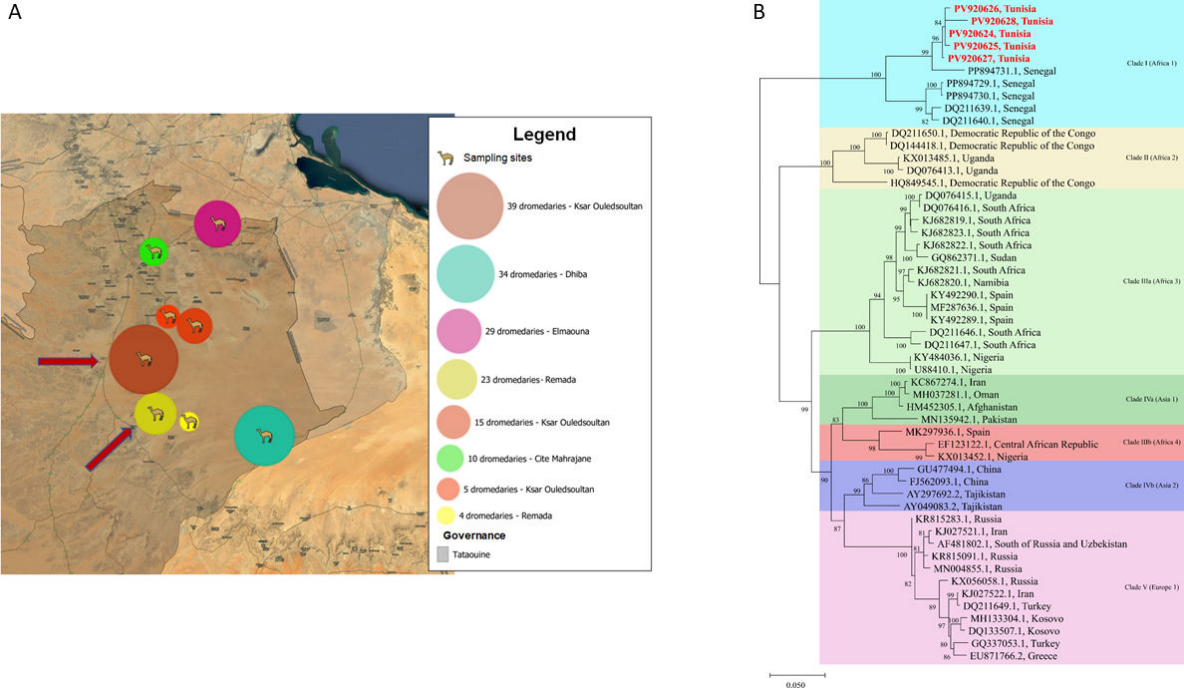
Local distribution of hosts. The arrows demarcate the locations where *Hyalomma* spp. testing positive for CCHFV were collected (**A**). The map was generated using QGIS software (version 3.38.2). Phylogenetic analysis of CCHFV in *Hyalomma* spp. ticks collected from dromedary camel, Tunisia, 2024 (**B**). Phylogenetic analysis of CCHFV S segment sequences was conducted in MEGA12 (https://www.megasoftware.net). The tree was constructed using the Maximum-Likelihood algorithm and Kimura2-parameter model. Results of the bootstrap test in 1,000 replicates are shown next to the branches. Only bootstrap values >80 are shown. Red and bold font indicates strains detected from Tunisia; other sequences are named by GenBank accession number and geographic origin. Clades of CCHFV, based on the S segment, are indicated; clade I (Africa 1; Senegal and West Africa), clade II (Africa 2; South and Central Africa), clade IIIa (Africa 3; West and South Africa), clade IIIb (Africa 4), clade IVa (Asia 1; Iran, Pakistan, and the Middle East), clade IVb (Asia 2; Kazakhstan, Tajikistan, and China), and clade V (Europe-1).

A total of 365 ticks were removed using fine forceps from parasitized dromedary camels across eight sampling localities in the Tataouine district of southern Tunisia (Fig. 1A).

Each tick was placed individually into a labeled sterile tube, and morphological identification was performed using taxonomic keys (6). All ticks were identified as belonging to the genus *Hyalomma*; however, due to the taxonomic challenges within this genus and the lack of molecular confirmation, we refer to these specimens as *Hyalomma* spp.

Each tick was homogenized using a mechanical tissue lyser (Qiagen Tissuelyser II). Nucleic acids were extracted from 200 µL of tick homogenate employing the MagNA Pure 96 System (Roche Applied Sciences), according to the manufacturer’s instructions. RNA was subject to one-step real-time RT-PCR for the detection of CCHFV, using the AgPath-ID™ One-Step RT-PCR Kit (Thermo Scientific). Previously reported primers and probes targeting CCHFV’s S segment were used (7). Five (1.37%) ticks tested positive (Table 1). Of these, three were collected from a female dromedary camel in the Ksar Ouled Soultan area and two from another in the Remada area (Fig. 1). CCHFV-positive samples were further subjected to PCR using primers CC-7F (5′-GTCAGGCCGTTCAGGAATAG-3′) and CC-10R (5′-ATTGCCCTTGACGTTGTAGG-3′), targeting a 935 bp region of the S segment of the viral genome (nucleotides 721–1655 of the strain IbAr10200, accession number U88410.1), (8) and amplicons underwent Sanger sequencing (Eurofins). The resulting sequences were subjected to analysis employing the Basic Local Alignment Search Tool (BLAST; http://blast.ncbi.nlm.nih.gov/Blast.cgi) alongside the GenBank database for verification of the viral identity. All sequences were deposited in the GenBank (Table 1).

**TABLE 1:** *Hyalomma* spp. ticks positive for CCHFV collected from dromedary camels in eight sites of Tunisia, 2024

Sample ID^*a*^	Stage	Host	Municipality	Geographic coordinates	Ct^*b*^	Isolate name/GenBank accession number
22A	Partially engorged male	Female dromedary camel/10-year-old	KOS	32.569075/10.447065	36.10	CCHFV/TUN/KOS/2024/22A/PV920625
22B	Partially engorged male				24.80	CCHFV/TUN/KOS/2024/22B/PV920626
22C	Unengorged female				36.38	CCHFV/TUN/KOS/2024/22C/PV920627
60A	Engorged male	Female dromedary camel/16-year-old	REM	32.366655/10.498951	35.11	CCHFV/TUN/REM/2024/60A/PV920628
60B	Partially engorged female				26.51	CCHFV/TUN/REM/2024/60B/PV920624

^*a*^ ID, identification.

^*b*^ Ct, cycle threshold.

Phylogenetic analysis showed that the CCHFV sequences clustered with those of Clade I (Africa 1) previously identified in Senegal and Algeria (Fig. 1B) (9).

This study substantiates the circulation of CCHFV within *Hyalomma* spp. infesting dromedary camels in Tunisia and provides the first phylogenetic characterization of CCHFV strains circulating in the country. Additionally, the detection of multiple positive ticks on individual animals supports the likelihood of infection in dromedary camels. This observation aligns with recent evidence of CCHFV exposure in dromedaries from neighboring Algeria (9), suggesting a broader role of these animals in the ecology and transmission of the virus. Phylogenetic analysis revealed that CCHFV strains belong to the Africa 1 clade, which is characteristic of West African and Algerian isolates and has also been identified in CCHFV-positive ticks from Spain and France. This finding underscores the importance of considering avian migratory routes in the epidemiology and geographical spread of CCHFV across the African continent and the Mediterranean basin.

## Data Availability

All data needed to evaluate the conclusions in the study are present in the paper. Sequences were deposited in GenBank with the following accession numbers: PV920624, PV920625, PV920626, PV920627, and PV920628.

## AFTER EPUB

*[This article was published on 23 September 2025 with typographical errors and a statement missing from Acknowledgments. The article was updated in the current version, posted on 11 May 2026.]*
